# Research on Crack Resistance of Foamed Rubber Asphalt Cold Recycled Mixtures Based on Semi-Circular Bending Test

**DOI:** 10.3390/ma18122684

**Published:** 2025-06-06

**Authors:** Zhen Shen, Shikun Wang, Zhe Hu, Xiaokang Zhao

**Affiliations:** 1 Overseas Department of CCCC Construction Group Co., Ltd., Beijing 110110, China; 2 School of Highway, Chang’an University, Xi’an 710064, China; zhaoxk@chd.edu.cn

**Keywords:** foamed rubber asphalt, cold recycled mixture, semi-circular bending test, digital image, cracking resistance

## Abstract

Foamed asphalt cold recycled mixtures can provide an effective approach for the reutilization of reclaimed asphalt pavement (RAP), but conventional asphalt foaming technology primarily exploits matrix asphalt as the raw material. To address this issue, this study explores rubberized asphalt with cold recycling technology to develop a foamed rubber asphalt cold recycled mixture (FRCM). The semi-circular bending (SCB) test was employed to investigate its cracking resistance. Load–crack mouth opening displacement (CMOD)–time curves under various temperatures were analyzed, and digital image technique was resorted to monitor crack propagation and growth rates. Fracture toughness, fracture energy, and flexibility index were compared with those of traditional foamed matrix asphalt cold recycled mixture (FMCM). The results show that, under the same test temperature, the FRCM exhibits slower crack propagation; larger peak load; and higher fracture toughness, fracture energy, and flexibility index in comparison with the FMCM. These improvements are more pronounced at low temperatures. For both mixtures, fracture toughness and fracture energy are decreased with increasing the temperature, while the flexibility index shows the opposite trend. The rigid zone accounts for a larger portion of fracture energy at low temperatures. The findings provide technical references for improving the cracking resistance of cold recycled asphalt layers using rubberized asphalt.

## 1. Introduction

The high-value and resourceful utilization of ground tire rubber and reclaimed asphalt pavement (RAP) has become one of the effective approaches for sustainable development in road transportation infrastructure, contributing to resource conservation and environmental protection [1,2]. Foamed asphalt cold recycling is a green pavement technology that utilizes reclaimed asphalt pavement materials at ambient temperature, incorporating a certain proportion of foamed asphalt, newly added virgin aggregates, cement, and mineral filler [3,4]. After mixing, compaction, and paving, it forms a structural layer that meets the performance requirements of recycled asphalt pavements. This technology has attracted extensive attention due to its advantages in cost-effectiveness, environmental friendliness, and energy conservation with emission reduction [5]. Currently, foamed asphalt cold recycled mixture is mainly applied in asphalt pavement rehabilitation, particularly in low-traffic flexible base courses and intermediate layers, with limited implementation under heavy traffic conditions [6]. As the conventional binder in foamed asphalt cold recycling, matrix asphalt suffers from deficiencies in pavement performance of recycled mixtures [7]. During service, issues such as raveling and cracking often occur due to insufficient high- and low-temperature stability, poor aging resistance, and inadequate moisture susceptibility, which severely affect the quality of recycled asphalt pavement projects [8,9]. Therefore, enhancing the road performance of foamed asphalt cold recycled mixture has become a bottleneck for large-scale application and promotion. In highway construction, researchers have attempted to convert waste tires into crumb rubber modified asphalt (CRMA) [10]. Relevant studies have shown that rubberized CRMA pavement exhibits excellent low-temperature crack resistance, aging resistance, and moisture stability, effectively reducing traffic noise and delaying pavement cracking and aging issues [11,12]. However, rubberized asphalt mixture requires higher mixing temperatures during production and construction due to its high viscosity, typically ranging from 170 °C to 190 °C, which is approximately 10–20% higher than the mixing temperatures for conventional asphalt mixtures (usually 150–160 °C), leading to massive carbon emissions and pollutant generation, posing health risks to construction workers [13]. If the advantages of foamed asphalt cold recycling and CRMA technology can be effectively integrated, it would not only reduce greenhouse gas emissions and environmental pollution but also improve the pavement performance of recycled pavement structural layers [14]. This approach could contribute significantly to achieving carbon neutrality and carbon peaking goals in the transportation domain, as the production of 1 t of foamed rubberized asphalt cold recycled mixture has been shown to reduce greenhouse gas emissions by approximately 45.13% and energy consumption by 51.38% compared to conventional hot-mix rubberized asphalt mixtures [15].

At present, the basic test methods for assessing the cracking resistance of asphalt mixtures include the semi-circular bending (SCB) test, thermal stress restrained stress test (TSRST), indirect tensile test (IDT), low-temperature bending beam test (BBT), and disc compact tension (DCT) test [16,17]. Based on fracture mechanics theory, the SCB test employs indicators such as fracture energy, fracture toughness, and flexibility index to assess the cracking characteristics of asphalt mixtures [18]. Additionally, the SCB test specimens are highly adaptable, and the internal stress distribution better reflects the actual stress conditions experienced by pavements [19]. The utilization of energy-based indicators for evaluating low-temperature cracking resistance is considered more comprehensive and reasonable, and the test results also show good correlations with in-service pavement performance [20]. Kavussi et al. [21] evaluated the SCB test results of foamed warm mix asphalt mixtures using the flexibility index, fracture energy, and cracking resistance index, revealing that the crack resistance of the mixtures improved with the incorporation of 30–50% RAP. Algraiti et al. [22] and Jeong et al. [23] investigated the influence of rejuvenators on the crack resistance of foamed asphalt cold recycled mixtures through the SCB test, demonstrating that directly adding rejuvenators into RAP materials significantly enhanced the cracking resistance of the recycled mixtures. Sabouri et al. [24] conducted the SCB test to assess the pavement performance of cold in-place recycling materials, and the results indicated that the fracture index value for energy effectively characterized the cracking resistance of the materials. Compared with the BBT, SCB specimens have a larger cross-sectional area and a slower loading rate, enabling a better representation of internal crack propagation characteristics in pavement structures [25]. In contrast to the TSRST, the SCB test offers superior applicability and operational simplicity, as specimens can be prepared using Marshall or rotary compaction methods or extracted directly from in-service pavements [26]. Furthermore, unlike the IDT and DCT tests, the SCB test effectively mitigates localized deformation damage induced by loading strips and allows for twice the number of specimens to be obtained under equivalent testing conditions [27].

Digital image technology provides an efficient and precise analytical tool for studying the fracture behavior of foamed rubber asphalt cold recycled mixture (FRCM) through the SCB test [28,29]. It offers advantages such as non-contact measurement, high measurement accuracy, and automation [30]. By using digital imaging techniques, the entire process of crack appearance, propagation, and final fracture on the surface of SCB specimens under loading can be captured in real time, revealing the damage evolution characteristics of the materials [31]. For example, Yang et al. [25] investigated the fracture process and damage behavior of fiber modified asphalt mixtures using the SCB test and employed the digital image technique to evaluate the interfacial fracture characteristics. The results showed that the three-dimensional distribution of fibers formed a spatial network inside the mixture, effectively delaying crack propagation and enhancing the cracking resistance. Zielinski et al. [20] employed the SCB test to evaluate the relationship between asphalt concrete aging conditions and its fracture performance. Digital image processing techniques were applied to assist laboratory experiments in interpreting the heterogeneity of asphalt mixtures, and digital image correlation was used to verify the accuracy of the test results. Similarly, Ren et al. [32] utilized digital image correlation techniques in the SCB test to study the fracture behavior of asphalt concrete, identifying critical stages of crack appearance and propagation. Hence, digital image technology enables more accurate analysis of crack propagation and the enhancement effects on cracking resistance, supporting the SCB test with more comprehensive results and facilitating the optimized design of asphalt mixtures for improved fracture performance [33]. Although digital image techniques have been applied in fracture testing of various pavement materials, their utilization in the SCB test for analyzing the fracture behavior of FRCM needs to be further explored.

Therefore, the main objective of this study is to evaluate the crack resistance performance of foamed rubber asphalt cold recycled mixtures (FRCM) and clarify the fracture mechanism using the SCB test combined with digital imaging analysis. The mixtures were prepared based on the specifications for RAP in asphalt pavement recycling technology. The fracture behavior of the SCB specimens was analyzed through the relationships among applied load, crack mouth opening displacement (CMOD), and time. Meanwhile, the crack propagation process was recorded using digital imaging equipment, and the crack growth rate was quantitatively evaluated. Furthermore, the fracture toughness, fracture energy, and flexibility index of FRCM were compared with those of FMCM under varying temperature conditions. The outcome contributes to providing technical evidence supporting the application of cold recycled mixture in durable and sustainable pavement structures.

## 2. Materials and Methods

### 2.1. Raw Materials

(1) Foamed asphalt

Two types of asphalt binders were utilized in this study: Donghai 70# matrix asphalt and activated rubberized asphalt. The foaming process was carried out using the WLB10S laboratory foaming device and the WLM30 twin-shaft horizontal mixer, both manufactured by Wirtgen (Windhagen, Germany). Based on previous research, the foaming characteristics of different asphalt were determined by adjusting the foaming temperature and water content in order to determine the optimal foaming conditions for rubberized asphalt. The specific procedures and steps followed the methodology described in the study by Zhao et al. [34]. The foaming performance of the asphalt was evaluated using two key indicators: the expansion ratio (the ratio of the maximum volume of foamed asphalt to the volume of unfoamed asphalt) and the half-life (the time required for foamed asphalt to decrease from its maximum volume to 50% of that value). The rubberized asphalt with superior foaming characteristics was selected, exhibiting an optimal foaming water consumption of 4.0% and an optimal foaming temperature of 165 °C, corresponding to the expansion ratio of 20.2 times and the half-life exceeding 30 s. In comparison, the Donghai 70# matrix asphalt was foamed at 160 °C with a water consumption of 2.9%, resulting in an expansion ratio of 15.5 times and a half-life of 11.8 s. The foaming characteristics of both types of foamed asphalt can meet the specification requirements of JTG/T 5521-2019 in China [35].

(2) RAP

The RAP was sourced from the surface layer of the asphalt pavement during a major rehabilitation project on a highway section in Shaanxi Province, China. A centrifugal extraction method was utilized to recover the aged asphalt from RAP materials, followed by testing three key asphalt properties and the dynamic viscosity at 60 °C. The results indicated that the softening point and dynamic viscosity increased, while penetration and ductility significantly decreased, suggesting that the original adhesive capability of the aged asphalt was essentially lost. Therefore, the RAP was treated as black aggregate in the mixture design. Gradation analysis was conducted on the extracted RAP aggregates. The contents of the aggregates passing through the 2.36 mm sieve size decreased by 20.4% compared to the lower limit, and the 2.36 mm sieve size increased by 3.5% compared to the upper limit. The overall gradation of the RAP materials did not meet the required gradation range for cold recycled mixtures. This indicates that, under long-term traffic loading, most of the coarse aggregates had fractured into medium and fine aggregates. As a result, additional newly added virgin aggregates were required to adjust the gradation of the recycled mixture to meet the standard. Moreover, technical performance tests were carried out on both the coarse and fine aggregates extracted from the RAP, as shown in Table 1.

(3) Newly added virgin aggregates and fillers

To meet the gradation design requirements of the foamed asphalt cold recycled mixture, newly added virgin aggregates and fillers were incorporated. Limestone mineral powder was used as the filler to form a denser aggregate skeleton structure of the cold recycled mixture. Additionally, 42.5 MPa Portland cement was added as an active filler to enhance the bonding strength and accelerate the initial curing of the mixture, thereby enhancing the mechanical properties of the cold recycled mixture.

### 2.2. Design and Preparation of the Cold Recycled Mixture

According to the JTG/T 5521-2019, the gradation of foamed asphalt cold recycled mixtures is categorized into fine-grained, medium-grained, and coarse-grained types. This study adopted the medium-grained gradation design for the foamed asphalt cold recycled mixture. Based on the gradation characteristics of the RAP, newly added virgin aggregates were introduced accordingly. Additionally, mineral filler and cement were incorporated into the mixture. The final gradation (by mass) of the cold recycled mixture was determined to consist of 63% RAP, 6% mineral powder, 1.5% cement, and 29.5% new aggregates. The detailed materials composition and gradation calculations result are shown in Table 2.

**Figure 1:**
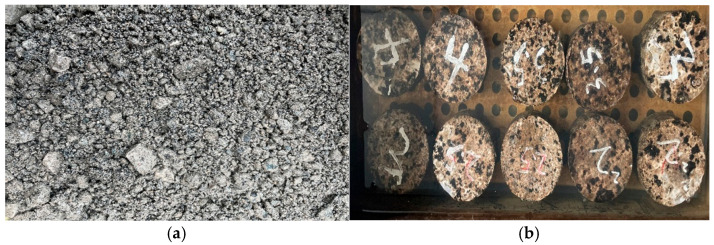
Foamed asphalt cold recycled mixture: (**a**) samples; (**b**) specimens.

The preparation procedure of the foamed asphalt cold recycled mixture (Figure 1a) is as follows: Firstly, the optimum moisture content of the cold recycled mixture without foamed asphalt was determined to be 6.1%, and the optimum mixing water content was 4.9% in accordance with the compaction method in JTG E40-2007 T0131 [36]. Then, a twin-shaft horizontal mixer was used to carry out a two-stage mixing process: Initially RAP, new aggregates, and fillers were mixed with water, followed by the addition of foamed asphalt, and each mixing stage lasted 60 s. After mixing, Marshall specimens (Figure 1b) were prepared using 75 times compaction on each side and cured at a constant temperature of 60 °C and demolded after cooling to room temperature. Six specimens were prepared for each mixture design, with three specimens used for the splitting test at 15 °C and the other three for the splitting test after 24 h of water immersion. Afterwards, the optimal foamed asphalt content was determined according to the peak values of the splitting strength at 15 °C and the tensile strength ratio (TSR) between dry and wet conditions. Ultimately, the optimum foamed asphalt content for both the FMCM and the FRCM was determined to be 3.0%. Under this condition, the dry splitting strengths were 0.48 MPa and 0.72 MPa, respectively, and the optimal dry–wet TSRs were 86.2% and 91.7%. No visible impurities such as oil or biological contaminants were observed during the preparation of the recycled mixtures.

At the optimal asphalt content of 3%, the FMCM only met the specification for the base and subbase layers under non-heavy and above traffic load conditions (splitting strength at 15 °C ≥ 0.40 MPa and TSR ≥ 75%). Notably, the FRCM can meet the surface layer requirements for the heavy and above traffic load levels (splitting strength at 15 °C ≥ 0.60 MPa and TSR ≥ 80%). Compared with FMCM, the splitting strength at 15 °C and TSR of FRCM increased by 50% and 6.4%, respectively. Therefore, the utilization of CRMA as the binder in cold recycled mixtures demonstrates significant performance advantages.

### 2.3. SCB Test

**Figure 2:**
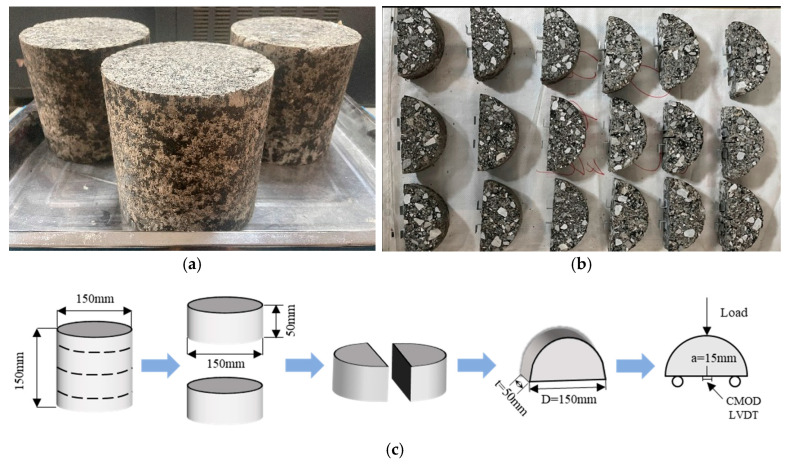
(**a**) Rotary compaction specimen; (**b**) SCB specimen; (**c**) specimen preparation process.

The preparation of SCB specimens began with forming cylindrical asphalt mixture samples using the rotary compaction method to achieve a size of Φ150 mm × h150 mm, as shown in Figure 2a. Due to the potential differences in compaction density between the top/bottom surfaces and the middle section, and considering that the surface layers are prone to damage, both the top and bottom layers (each 25 mm thickness) were removed using a cutting machine to ensure consistency. The central portion of the specimens were then cut to obtain four semi-circular specimens (Φ150 mm × h50 mm). A 15 mm length and 1.5 mm width notch was introduced perpendicular to the flat edge of each specimen and aligned with the center to facilitate controlled crack propagation during testing. After specimen preparation, the samples were cured for 28 days under standard conditions at 20 ± 5 °C and 40–70% relative humidity. The SCB test was conducted on a MTS universal testing machine, with three replicate specimens per group, to ensure result reliability. The semi-circular bending specimens and its preparation process are shown in Figure 2b,c.

**Figure 3:**
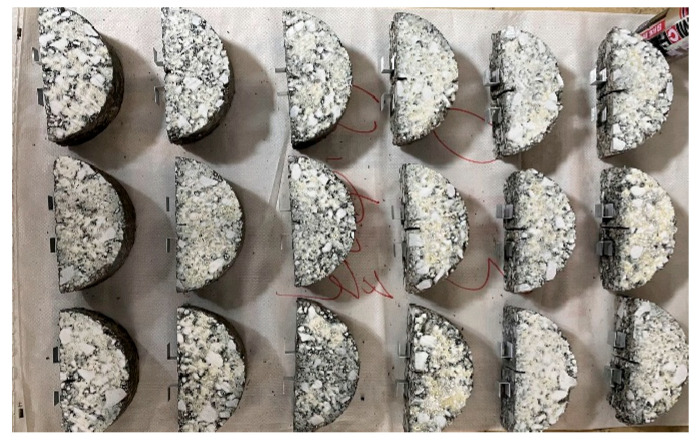
SCB specimens after spray dyeing.

To more clearly highlight the crack propagation procedure of the specimens, the surface of the SCB specimens was sprayed with white paint, as shown in Figure 3. The CMOD of the SCB specimens was measured using a YYU-10/25 electronic extensometer manufactured by Steel Research and Testing Technology Co., Ltd. (Beijing, China), with a measurement range of 10 mm. For accurate attachment, 23/17 staples were adhered to both sides of the notch at the bottom of the SCB specimens using strong adhesive glue. The clips of an extensometer were then secured onto the staples using rubber bands, ensuring stable and reliable displacement measurements during the test.

**Figure 4:**
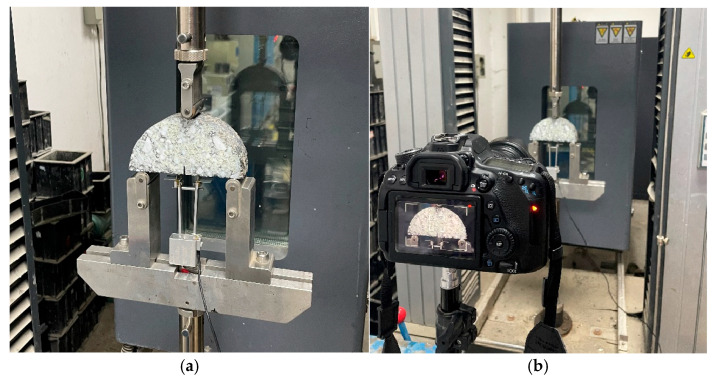
SCB test process: (**a**) extensometer installation and specimen placement; (**b**) camera takes pictures to record the cracking process.

Considering that specimens are prone to brittle fracture and rapid crack propagation at excessively low temperatures, making it difficult to compare the performance of the two types of recycled mixtures, the test temperatures were set at −5 °C, 10 °C, and 25 °C. To ensure thermal equilibrium, the specimens were placed in a MTS temperature-controlled chamber for 6–8 h at each testing temperature prior to the experiment, and the time from the removal of the specimens from the chamber to the start of the SCB test was strictly controlled. The SCB testing procedure is shown in Figure 4. The support points at the bottom of the specimens were aligned to ensure the specimen was centered both laterally and longitudinally, with the distance set at 0.8 diameter (120 mm). Loading was applied using a MTS universal testing machine, with the loading head vertically aligned with the central notch of the specimens. This alignment ensured that the loading direction coincided with the notch of the specimen. The loading rate was set to 2 mm/min, as a slower loading speed can promote crack propagation and damage evolution. In addition, a high-resolution camera was employed to record the loading process, with the camera axis positioned perpendicular to the specimen surface, providing a clear visualization of the crack propagation and fracture characteristics.

## 3. Results and Discussion

### 3.1. Analysis of the Fracture Process and Characterization of the Mixtures Based on the SCB Test

#### 3.1.1. Load–CMOD–Time Curve

**Figure 5:**
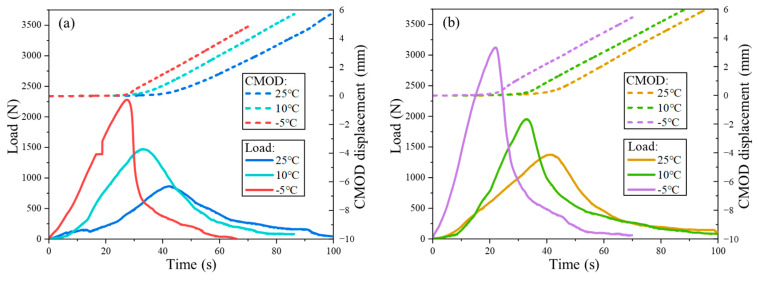
Load and CMOD–time curves at different temperatures: (**a**) FMCM; (**b**) FRCM.

This study plotted the load–time and CMOD–time curves for FMCM and FRCM at different temperatures, as shown in Figure 5. It can be observed that, for both mixtures, the load–time relationship exhibited a typical trend of increasing to a peak followed by a decline, indicating a complete loading and failure process. Under the 25 °C test conditions, both types of asphalt mixtures mainly exhibit viscoelastic behavior, with the load reaching its peak after more than 40 s. After reaching the peak load, visible microcracks gradually began to appear at the bottom notch of the SCB specimen, and the CMOD started to change. After the peak load, the microcracks progressively extend to form cracks, the strength of the mixture begins to degrade, and the load drops to zero. Eventually, cracks propagated through the entire specimen, leading to ductile failure. Under the 10 °C condition, the peak load for both mixtures is greater than at 25 °C, and the peak load occurs earlier, between 30 and 40 s. This can be attributed to the increase in stiffness of the mixtures at lower temperatures. When the test temperature was further dropped to −5 °C, a significant increase in the peak load was observed, and the peak load occurred earlier around 20–30 s. After the peak load, the load decreased rapidly, indicating that the specimen experienced brittle fracture behavior. The load–displacement curve is not completely smooth. This is because microcracks generated inside the specimen accumulate along the edges of the aggregates, leading to macroscopic microcracks and abrupt load fluctuations.

The comparison of the fracture processes of the two types of asphalt mixtures reveals that, under the same temperature, the critical failure load of the FRCM is significantly higher than that of the FMCM. This can be attributed to the stronger adhesion property of rubberized asphalt, which results in a more robust bond with the aggregates. An analysis of the CMOD–time curves for the two mixtures shows that, before the peak load, the CMOD remains essentially unchanged and close to 0, indicating no significant deformation in the SCB specimen. After the peak load, the CMOD begins to increase, showing a linear growth trend. Moreover, the CMOD evolution curves of the two mixtures under different temperatures are nearly parallel, indicating a similar rate of crack opening. This suggests that the resistance mechanisms of the FRCM and FMCM in the crack propagation process are similar, but the FRCM demonstrates a greater crack resistance, with its peak load being approximately 36.4% and 33.3% higher than that of the FMCM at −5 °C and 10 °C.

#### 3.1.2. Crack Fracture Process and Propagation Rate

**Figure 6:**
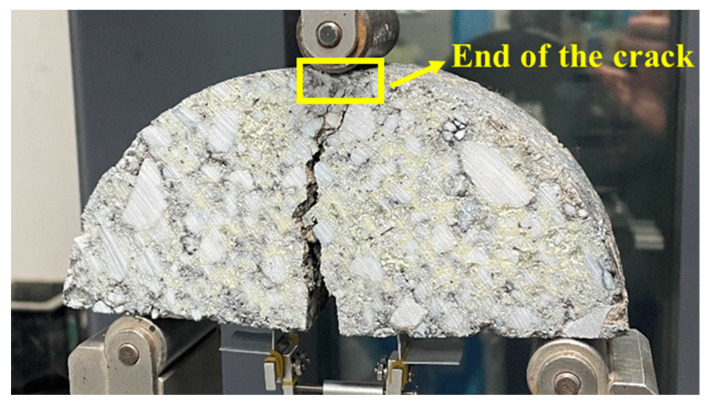
Fracture state of the SCB specimen.

According to different stress states and fracture characteristics, crack propagation can be classified into three basic modes: crack opening mode (I), crack sliding mode (II), and tearing mode (III). The fracture states of the SCB specimen after loading are shown in Figure 6. Under the applied load, the tensile stress is generated at the specimen bottom, and cracks initially form at the tip of the notch. The crack then propagates upwards along the original notch direction, eventually penetrating through the entire specimen. Therefore, the fracture mode is identified as the crack opening mode (I). The end of the crack was defined as the moment when the primary crack extended through the entire ligament of the SCB specimen, accompanied by a significant drop in load (approaching zero) on the load–CMOD–time curve. This was further confirmed visually through digital imaging. Figure 6 also shows a representative cracked specimen with the end of the crack indicated.

**Figure 7:**
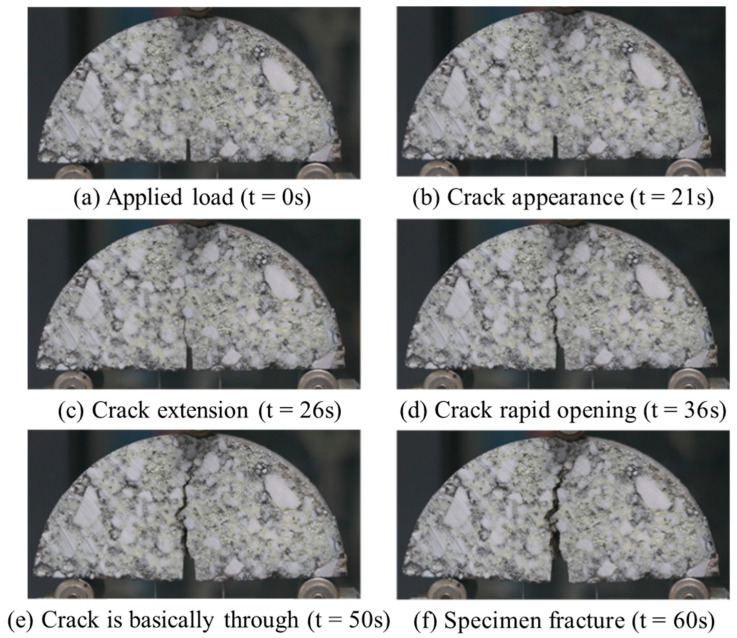
Foamed rubber asphalt cold recycled mixture specimen crack propagation fracture process at −5 °C.

Furthermore, a Canon digital device with high resolution and a sufficient frame rate was utilized during the experiment to continuously record the entire cracking development process of the SCB specimen. The cracking process of the FRCM specimen under the −5 °C test condition at different time points was recorded, as shown in Figure 7.

Figure 7 demonstrates that, during the loading phase, no visible changes occurred on the surface of the specimen. After 21 s of loading, internal microcracks had already formed, and fine cracks began to appear on the surface. As the loading continued, the load reached its maximum value and began to decay, and the crack gradually expanded. The crack propagation mainly occurred at the bonding interface between the aggregates and the asphalt, and the cracks tended to circumvent the surfaces of the larger aggregates. As the loading head continued to descend, the crack mouth opening displacement continued to increase. Around 60 s, the crack had propagated through the entire specimen, signifying that the SCB specimen had fractured.

In order to better understand the crack propagation procedure of the SCB specimens under a load, the crack propagation distance per unit time was calculated. The crack propagation velocity was determined using the following formula, as shown in Equation (1):

(1) $$v=\frac{\Delta s}{\Delta t}$$

where *v* is the crack propagation speed (mm/s); Δ*s* is the crack propagation path length (mm); Δ*t* is the crack propagation time (s).

**Figure 8:**
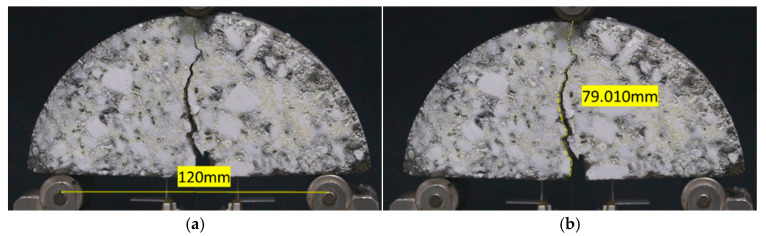
Crack propagation path calibration and measurement for the SCB specimen: (**a**) size calibration; (**b**) path measurement.

Potplayer V1.7 software was resorted to capture real-time video frames during the specimen loading process. The extracted images were then imported into ImageJ V1.8 software for path calibration and crack length measurement, as shown in Figure 8. The distance between the two bottom support points was selected as the calibration basis, and the scale was set to 0.8 in diameter (120 mm). The Segmentedline tool in ImageJ was employed to trace the crack propagation location, and the measured path length was output as the crack propagation length at the time of the SCB specimen fracture. By displaying the real-time video, the time from crack appearance to specimen fracture was recorded, and the crack propagation rate was calculated.

**Figure 9:**
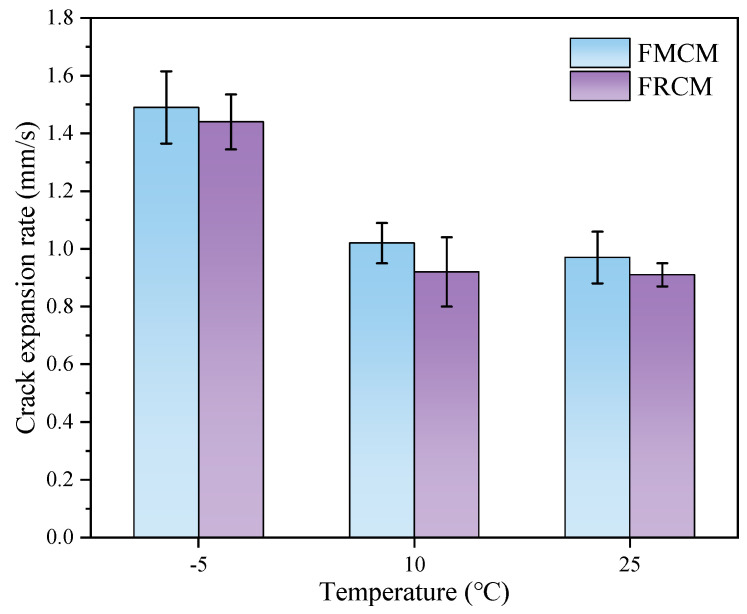
Crack propagation rate of the SCB specimens.

Figure 9 shows the crack propagation rate test results for various specimens at different temperatures. The results indicate that, when the test temperature decreases, the crack propagation rate of both types of cold recycled mixtures gradually increases. This is attributed to the fact that asphalt mixtures tend to exhibit brittle behavior at lower temperatures, thus leading to faster crack propagation. However, the crack propagation rate of FRCM shows almost no difference between 10 °C and 25 °C, which may be attributed to the fact that the fracture process is dominated by material toughness in this temperature range. In addition, rubber particles in the FRCM play a significant toughening role and exhibit strong temperature adaptability. Within the temperature range of 10 °C to 25 °C, the rubber particles can maintain good elasticity, continuously absorbing and dissipating part of the energy during crack propagation, thereby stabilizing the crack propagation rate. Moreover, the crack propagation rate of the FMCM was higher than that of the FRCM for all three test temperatures, indicating that the FRCM exhibits better toughness and crack resistance. This can be attributed to the reality that rubber particles can reduce the stress concentration at the crack tip, resulting in higher resistance to crack propagation in the recycled mixture.

### 3.2. Analysis of the Crack Resistance of the Mixtures Based on the SCB Test

#### 3.2.1. Fracture Toughness

The fracture toughness *K_IC_* corresponds to the stress intensity factor *K_I_* at the peak load *Pc*, and it can be utilized to characterize the stress distribution at the crack tip during the cracking process of the asphalt mixtures specimens. When the stress intensity factor of the material *K_I_* reaches the fracture toughness *K_IC_*, visible cracks begin to form. As the loading continues, these cracks rapidly propagate. Therefore, a larger *K_IC_* value indicates a higher stress intensity at the maximum load, meaning the asphalt mixture absorbs more energy during the fracture procedure. This results in better resistance to crack propagation. The equations for calculating the fracture toughness *K_IC_* can be seen as follows:

(2) $$K_{IC}=Y_{I(0.8)}\sigma_{0}\sqrt{\pi a}$$

(3) $$Y_{I(0.8)}=4.782+1.219\left(\frac{a}{r}\right)+0.063e^{7.045\left(\frac{a}{r}\right)}$$

(4) $$\sigma_{0}=\frac{P_{c}}{2rt}$$

where *K_IC_* is the fracture toughness (MPa·m^0.5^); *Y_I_*_(0.8)_ is the standard stress intensity factor, dimensionless; *P_c_* is the peak load (mN); σ_0_ is the stresses corresponding to the peak load (kN); *a* is the depth of the cut at 0.015 m; *r* is the radius of the SCB specimen at 0.075 m; *t* is the thickness of the SCB specimen at 0.05 m.

**Figure 10:**
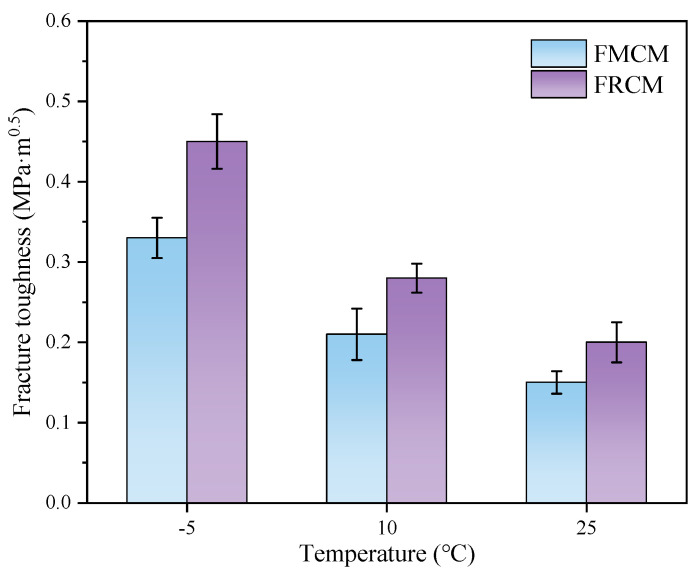
Fracture toughness of the specimens at different temperatures.

The fracture toughness results for FMCM and FRCM at three different temperatures are shown in Figure 10. The results indicate that the fracture toughness of both cold recycled mixtures is highly influenced by temperature. The fracture toughness of both mixtures increases when the temperature decreases. This trend is especially evident between 10 °C and −5 °C, where the fracture toughness rises significantly by more than 50% for FRCM and FMCM, with increases of 60.7% and 57.1%, respectively. This can be ascribed to the reality that a decrease in temperature has a stiffening effect on asphalt, leading to a higher load-bearing capacity and higher fracture toughness of the asphalt mixture.

Moreover, the fracture toughness of both cold recycled mixtures at −5 °C is 2.25 times and 2.2 times higher than at 25 °C, respectively, indicating that more energy is required for fracture propagation at lower temperatures. Notably, at the same temperature, the fracture toughness of the FRCM is consistently higher than that of the FMCM, with this advantage being particularly prominent at low temperatures. Hence, the results of the fracture toughness suggest that the FRCM has better low-temperature crack resistance than the FMCM. However, it should be noted that fracture toughness depends on the magnitude of the peak load and does not account for the displacement factor during the fracture of SCB specimens. As a result, it may not fully reflect the comprehensive performance differences between various asphalt mixtures.

#### 3.2.2. Fracture Energy

Fracture energy *G_f_* can also be utilized to assess the cracking resistance of asphalt mixtures. It is the ratio of the fracture work to the crack propagation area, and the larger *G_f_* indicates the better cracking resistance of the mixture. The specific calculation is as follows:

(5) $$G_{f}=\frac{W_{f}}{A_{lig}}$$

where *G_f_* is the fracture energy (J/m^2^); *W_f_* is the fracture work (J), $W_{f}=\int pd(u)$; A_lig_ is the crack propagation area (m^2^), $A_{lig}=(r-a)t$.

In addition, the fracture work refers to the work done by the external force on the SCB specimens of the cold recycled mixture. It can be calculated as the integral of the load–vertical displacement curve along the x-axis, from the onset of deformation to the point where the residual load drops to 100 N. The specific calculation formula is as follows:

(6) $$W_{f}=\int_{0}^{u_{0}}P_{1}(u)du+\int_{u_{0}}^{u_{final}}P_{2}(u)du$$

where *P*_1_(*u*) is the pre-peak fitting curve; *P*_2_(*u*) is the post-peak fitting curve; $u_{0}$ is the displacement of the peak load (mm); $u_{\mathrm{final}}$ is the displacement of the 0.1 kN residual load (mm).

**Figure 11:**
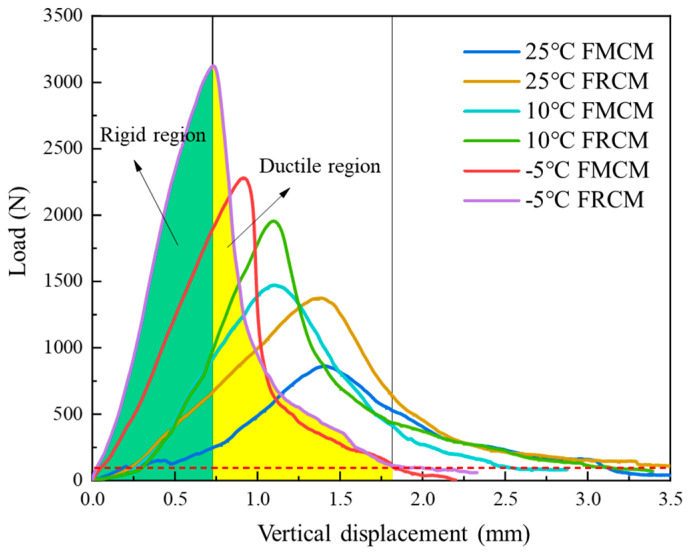
Load–vertical displacement curve.

As shown in Figure 11, the peak load serves as a boundary, dividing the curve into two regions in the load–vertical displacement curve: the rigid and the ductile regions. In this study, the load–displacement curve was fitted using software Origin 2024, and the area enclosed by the fitted curve and the x-axis was integrated to calculate the fracture work of the cold recycled mixtures under different temperatures. Then, the fracture energy was obtained by calculating the ligament area based on the specimen thickness and ligament length. Three parallel tests were performed under each test condition, and the average fracture energy value was taken to ensure the reliability of the experimental data.

**Figure 12:**
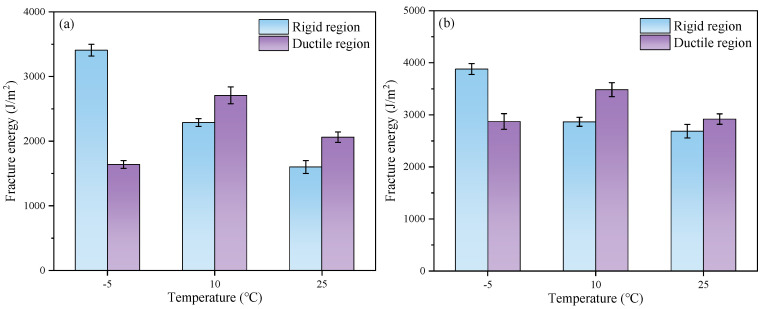
Fracture energy of the specimens at different temperatures: (**a**) FMCM; (**b**) FRCM.

The fracture energy of the FMCM and FRCM at different temperatures is shown in Figure 12. Compared to the matrix asphalt, rubberized asphalt can effectively enhance the cracking resistance of the cold recycled mixture. As the temperature increases, the fracture energy in the rigid region of both mixtures decreases. Compared with the FMCM, the fracture energy in the rigid region of the FRCM is increased by 13.8%, 25.3%, and 67.8%, respectively, at the test temperatures of −5 °C, 10 °C, and 25 °C, and the fracture energy in the ductile region is increased by 75.3%, 28.6%, and 41.5%, respectively.

**Figure 13:**
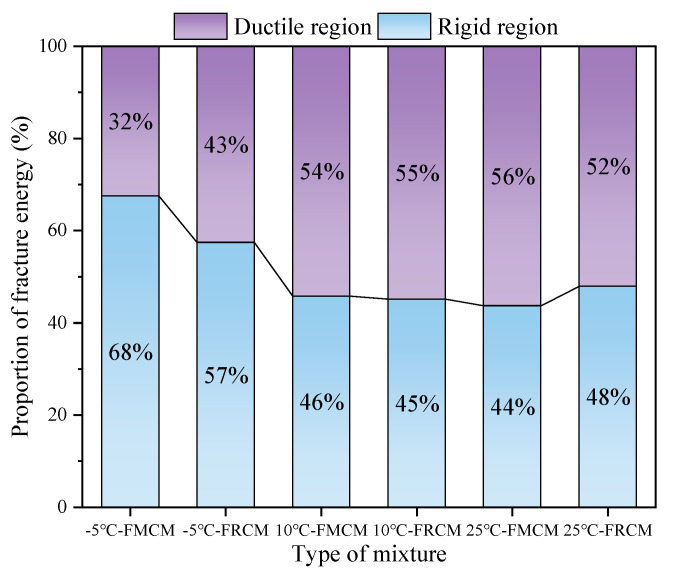
Proportions of the fracture energy in the rigid and flexible zones.

Additionally, the fracture energy in the rigid and ductile regions was calculated separately, and the proportion of each region is shown in Figure 13. The findings indicate that the trend of fracture energy in the rigid region of both asphalt mixtures is consistent with the overall fracture energy trend. Under the −5 °C low temperature, the SCB specimen exhibits a high peak load and is prone to brittle failure. Therefore, after reaching the peak load, the load decreases rapidly, resulting in a larger proportion of fracture energy in the rigid region before the peak load. When the test temperature increases, the stiffness of the asphalt mixture decreases, and its ductility increases, causing the proportion of fracture energy in the rigid region to decrease. At low temperatures, the matrix asphalt exhibits a higher degree of brittleness compared to rubberized asphalt, which also contributes to a significantly smaller proportion of fracture energy in the rigid region for the FRCM compared to the FMCM. However, due to the higher strength of the FRCM, its fracture energy in the rigid region is still larger than that of the FMCM. Under the 10 °C and 25 °C test conditions, there is little difference in the stiffness of the FRCM and FMCM, and the proportions of fracture energy in the rigid region for both cold recycled mixtures are quite similar.

#### 3.2.3. Flexibility Index

The fracture energy is insufficient to evaluate asphalt mixtures with the same fracture energy and different load–displacement characteristics [37]. The flexibility index can be calculated by the ratio of fracture energy to the slope at the inflection point of the load–displacement curve after the peak load, which can effectively evaluate the crack propagation process. The slope at the inflection point after the peak load can effectively characterize the unloading rate during the crack appearance to full propagation and can also reflect the extent of brittle fracturing during the unloading process. The specific calculation formula is as follows:

(7) $$FI=\frac{G_{f}}{\left|m\right|}*A$$

where *FI* is the flexibility, dimensionless; *m* is the value of slope at the inflection point of the load displacement curve after the peak load; *A* is the calibration factor coefficient, and the default value is 0.01 (kN/mm^2^).

The flexibility index results of the specimens at different test temperatures are shown in Table 3. The results indicate that the flexibility index increases significantly when the temperature increases. A higher value of the flexibility index represents slower crack propagation and stronger resistance to cracking. At low temperatures, the specimens exhibit more brittle behavior, so the crack propagation rates of the FMCM and FRCM are nearly identical, and the values of the flexibility index show little difference. When the test temperature rises from −5 °C to 10 °C, and then from 10 °C to 25 °C, the growth rates (slopes) of the flexibility index for the FMCM are 0.582 and 1.369, respectively. For the FRCM, the growth rates (slopes) are 1.678 and 2.201, respectively. The average growth rate of the flexibility index for the FRCM is approximately twice that of the FMCM as the temperature increases from −5 °C to 25 °C. Therefore, it can be concluded that, compared to the FMCM, the FRCM exhibits slower crack propagation and better resistance to cracking damage. This is consistent with the results obtained from the fracture toughness and fracture energy indicators. Furthermore, as the temperature increases, the improvement in mixture toughness and the enhancement of the crack resistance performance become more pronounced in the FRCM.

## 4. Conclusions

This study combined two environmentally friendly asphalt cold recycling and rubberized asphalt technologies to prepare the FRCM and systematically evaluated its cracking resistance compared to traditional FMCM through the SCB test with digital imaging equipment under different temperature conditions. The following conclusions were drawn from the study:

(1) The FRCM can satisfy the technical design requirements of the surface layers under medium and heavy traffic, while the FMCM only meets the requirements for the base layers. At all three test temperatures, the peak load of the FRCM is 33.3–36.4% higher than that of the FMCM, indicating an improved load-bearing capacity.
(2) Compared to the FMCM, the FRCM exhibits a slower crack propagation rate and greater flexibility index, indicating better resistance to crack growth. As the temperature increases from −5 °C to 25 °C, the average growth rate of the flexibility index for the FRCM is approximately twice that of the FMCM.
(3) The fracture toughness of both the FRCM and FMCM increases as the temperature decreases, with a particular rise observed between −5 °C and 10 °C. In this range, the fracture toughness of the FRCM and FMCM increases by 60.7% and 57.1%, respectively, indicating enhanced crack resistance at lower temperatures.
(4) The fracture energy of the FRCM is higher than that of the FMCM under all three test temperatures, indicating better crack resistance. The proportion of fracture energy in the rigid region exceeds 50% at low temperatures.

This study utilizes digital image techniques to assist the SCB test, which is currently limited to performance validation in practical engineering and macroscopic-scale evaluations. Future research may establish a multiscale evaluation for the FRCM by integrating the macroscopic mechanical performance with microscopic structural characterization and further investigate its high-temperature rutting resistance, moisture susceptibility, and durability. In addition, developing a carbon footprint assessment framework and conducting a cost–benefit analysis would further promote the advancement of this environmentally friendly and sustainable pavement technology.

## Figures and Tables

**Table 1:** Technical indicators of the RAP.

Materials Testing Programs			Measured Value	Requirements
RAP	Moisture content (%)		1.03	≤3%
	Asphalt content (%)		3.37	>3%
Coarse aggregates	Needle content (%)	Grain size < 9.5 mm	8.1	≤15%
		Grain size > 9.5 mm	3.9	≤20%
	Crushing value (%)		25.1	surface layer ≤ 26%
				Other layers ≤ 28%
Medium and fine aggregates	Angularity		50%	≥40%

**Table 2:** Materials composition and gradation.

Sieve Size (mm)	Percent Passing (%)					
	63% RAP	6% Mineral Powder	1.5% Cement	29.5% New Aggregates	Synthetic Gradation	Specification Requirements
26.5	100	100	100	100	100	100
19	98.1	100	100	95.2	97.4	85.0–100
16	96.8	100	100	78.2	91.6	-
13.2	94.1	100	100	60.3	84.6	-
9.5	82.8	100	100	48.5	74.0	55.0–80.0
4.75	42.3	100	100	42.6	46.7	35.0–60.0
2.36	19.9	100	100	38.1	31.3	25.0–45.0
1.18	10.0	100	100	35.4	24.2	-
0.6	5.5	100	100	25.1	18.4	-
0.3	2.0	100	100	16.7	13.7	8.0–22.0
0.15	0.9	100	100	8.0	10.4	-
0.075	0.3	89.5	99.2	2.2	7.7	4.0–12.0

**Table 3:** Flexibility index of the SCB specimens at different temperatures.

Type of Mixture	Flexibility Index		
	−5 °C	10 °C	25 °C
FMCM	1.26	9.99	30.52
FRCM	4.09	29.26	62.28

## Data Availability

The original contributions presented in this study are included in the article. Further inquiries can be directed to the corresponding author.
